# Supplementary material for: Whole-Genome Shotgun Sequencing from Chicken Clinical Tracheal Samples for Bacterial and Novel Bacteriophage Identification
Source: Vet Sci. 2025 Feb 12;12(2):162. doi: 10.3390/vetsci12020162 (PMC11861695; doi:10.3390/vetsci12020162)
Supplement: Supplementary file 1 [file vetsci-12-00162-s001.zip › vetsci-3438182-supplementary.pdf]

Supplementary material

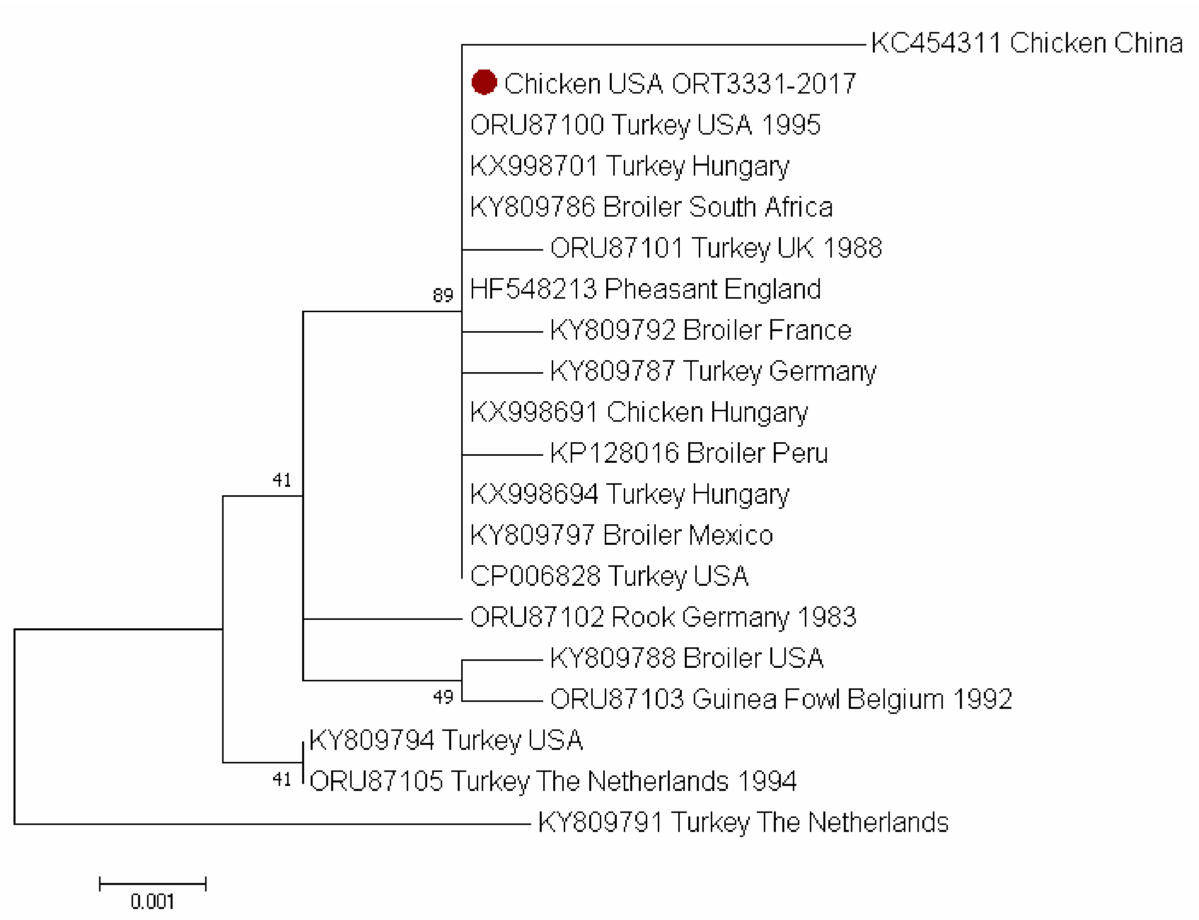

Suppl. Fig. S1. Phylogenetic tree based on 16S rRNA gene sequences constructed by MEGA7 using Maximum Likelihood method based on the Hasegawa-Kishino-Yano model. The bootstrap analysis of 1000 replicates were performed to estimate the confidence of tree topologies. The analysis involved 20 nucleotide sequences. All positions containing gaps and missing data were eliminated. There were a total of 1334 positions in the final dataset. The chicken ORT assembled this study (ORT3331-2017) is mark in red.
